# Population-based cardiovascular cohort studies in Uppsala

**DOI:** 10.1080/03009734.2018.1515282

**Published:** 2018-10-03

**Authors:** Lars Lind

**Affiliations:** Department of Medical Sciences, Uppsala University, Sweden

**Keywords:** Cardiovascular, cohort study, heart, longitudinal, vessels

## Abstract

The first population-based cohort study in Uppsala with the aim to study cardiovascular disease was initiated in 1970 (ULSAM). This cohort of 2300 middle-aged men has since then been followed in a longitudinal fashion for almost 50 years. This study has been followed by the PIVUS study, investigating 1000 men and women at ages 70, 75, and 80. A very detailed examination has also been performed in 500 subjects aged 50 years, the POEM study. In recent years, a high-throughput study conducted in 13000 subjects has also been performed, named EpiHealth. Uppsala also collects data in 5,000 subjects in the nationwide SCAPIS study. Taken together, these cardiovascular-oriented studies constitute a very rich source for cardiovascular epidemiological research in Uppsala. This review summarizes the design of these studies and highlights some of the important results published based on data from these studies.

## Introduction

Framingham Heart Study (FHS) was initiated in 1948 to study the natural course of cardiovascular disease (CVD) and its risk factors. Since then this cohort study has been the leading epidemiological CVD study. Inspired by FHS, Gösta Tibblin and co-workers started the study of ‘Men born 1913’ in 1963 in Gothenburg. At that time, it was recognized that coronary heart disease mainly was a disease of men, and therefore only men were included.

## The ULSAM study

Inspired by these two milestone studies in the USA and in Sweden, Hans Hedstrand and co-workers at the Department of Medicine at Uppsala University Hospital initiated in 1970 a cohort study of 2,322 men all aged 50 years that was later named ‘the Uppsala Longitudinal Study of Adult Men’ (www.pubcare.uu.se/ulsam) (1). The aim from the beginning was to investigate risk factors for CVD, and then to randomize the individuals to a more intense intervention or not, in order to evaluate if future CVD could be prevented. This aim of the study was not really fulfilled, but this cohort study has served as the basis for more than 40 PhD theses and 350 publications during the years.

Unique features of the initial investigation at age 50 years were an intravenous glucose tolerance test with frequently sampled insulin determinations, to determine glucose-stimulated insulin secretion, and a detailed characterization of the fatty acid profile in cholesterol esters, a marker of dietary fat quality intake.

Following the first examination cycle, Hans Lithell at the Department of Geriatrics took over the responsibility of the study. Although an examination was performed at age 60, it was the examination at age 70 that put ULSAM on the international scene. Apart from traditional CVD risk factors, an oral glucose tolerance test (OGTT) with insulin determinations was performed together with the hyperinsulinemic euglycemic clamp that allowed an evaluation of both insulin secretion and sensitivity. This is still the largest effort in a single study to characterize these two major determinants of diabetes development. Other unique features of the 70-year examination cycle were an echocardiographic examination including determinations of left ventricular geometry and function, as well as skeletal muscle biopsies to assess the fiber composition in half of the sample. Based on the ‘development origin of adult disease (DOAD)’ hypothesis also the birth weight was included, and a 24-h ambulatory blood pressure measurement was performed.

At the 70-year examination, and even more in the following years, also diseases other than CVD have been studied, and data from dietary records, cognitive function tests, and measurements of bone mineral content by dual X-ray absorptiometry (DXA) and bone fractures have been studied (2).

Major findings published over the years regarding CVD in ULSAM include the following: proinsulin is an important risk factor for CVD (3); left ventricular mass determined both at echocardiography and at ECG are additive risk factors for CVD (4); insulin sensitivity is a risk factor also for stroke (5) and heart failure (6); metabolic syndrome is a risk factor for CVD (7); atrial fibrillation is a risk factor for poor cognitive function (8); isolated ambulatory hypertension is a risk factor for CVD (9); increase in fasting glucose during treatment with beta-blockers increased the risk of myocardial infarction (10); a set of biomarkers predicted cardiovascular (CV) mortality (11); obesity without metabolic risk factors, so-called metabolically healthy obesity, was related to premature CVD (12); and a certain fatty acid profile, including high proportions of saturated fat and a low proportion of linoleic acid, was related to incident myocardial infarction (13).

**Figure 1.**
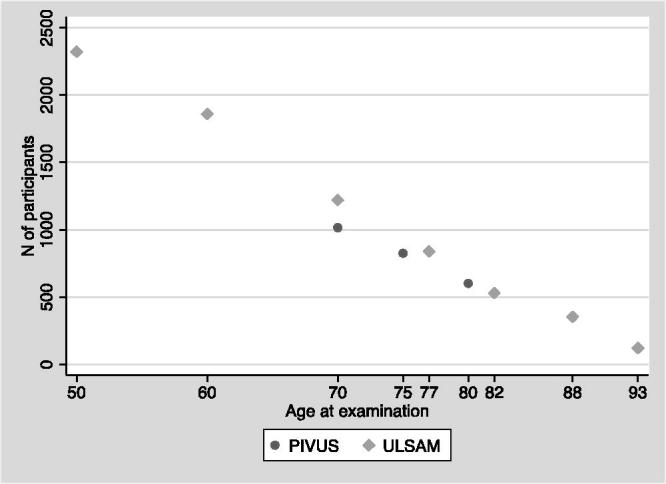
Number of participants at each examination cycle in the ULSAM and PIVUS studies.

The major strength of the ULSAM study is the many re-examinations performed, which allows for collection of longitudinal trends of measured variables as well as for collection of incident cases of a number of diseases, not only CVD, over more than 40 years. Figure 1 shows an overview of the number of participants at each examination cycle.

## The PIVUS study

With the major aim to study if measurements of endothelial function would add predictive power on top of traditional risk factors regarding CVD, the Prospective Study of the Vasculature in Uppsala Seniors (PIVUS) study was initiated in 2001 (www.medsci.uu.se/pivus) (14).

In this study, all 1,016 participants were 70 years old, and 50% of the sample were women. Apart from endothelial function the vasculature was characterized by arterial compliance and carotid artery ultrasound measurements to evaluate atherosclerosis, and total atherosclerotic burden by magnetic resonance angiography. The heart was examined by echocardiography and magnetic resonance imaging (MRI) with late enhancement.

Fat distribution was characterized by DXA and abdominal MRI. Dietary data were recorded, and FA composition in cholesterol esters were determined.

At age 75, the sample was reinvestigated, and at this time MRI of the brain and three cognitive function tests were performed. These investigations were also repeated at age 80 (see Figure 1 for numbers of participants). Incident cases of CVD have been recorded over 10 years, and will be updated after 15 years.

More than 250 papers have been published, and more than 20 PhD students have had PIVUS data in their thesis. Major results being published showed that endothelial function in the forearm resistance vessels, but not in the brachial conduit artery, was related to future CVD (15); silent myocardial scars were more common than previously thought (16); silent myocardial scars were related to cerebral infarcts in women only (17); and troponin I levels were related to both CV and non-CV mortality (18).

Since the results in many studies probably are false positive findings and cannot be reproduced by others, we have in a number of studies used data from both ULSAM and PIVUS to evaluate the use of biomarkers. By this approach, it has been shown that plasma levels of endostatin (19) and cathepsin S (20) are related to mortality in these two independent samples.

Since the first genome-wide association studies (GWAS) were published some 10 years ago, ULSAM and PIVUS have participated in a number of the big consortia trying to find the genetic architecture of obesity, diabetes, coronary heart disease, and stroke. There are two major conclusions from these studies. First, the top gene findings, the FTO gene, the TF gene, and the 9.21 loci, were largely unknown gene regions before the GWAS era and could therefore not be found by a candidate gene approach. Second, in none of these outcomes was a single or a handful of genes of major importance; rather, hundreds of genes have a small impact each.

During the recent years, ULSAM and PIVUS have put a large effort into proteomics and metabolomics and published that low levels of lyophosphatidylcholine (18:2) are associated with a high risk of myocardial infarction (21), that certain bile acids are related to diabetes risk (22), and have identified a number of proteins being associated with carotid atherosclerosis (23), incident stroke (24), and atrial fibrillation (25).

The PIVUS study has become famous in the field of environmental research in that we have measured >50 different environmental contaminants in plasma and found, amongst other things, that persistent organic pollutants, such as PCBs, perfluorinated compounds, and pesticides, are related to atherosclerosis (26,27) and incident stroke (28).

The major strength of the PIVUS study is the detailed characterization of the cardiovascular system at repeated examinations.

## The POEM study

The population-based Prospective investigation of Obesity, Energy and Metabolism (POEM) study was conducted in inhabitants of Uppsala, Sweden, all aged 50 years. Between October 2010 and October 2016, 502 individuals were investigated (50% women). The primary aim was to explore the links between obesity and CVD.

A great number of markers of subclinical CV disease (three tests of endothelial function, three tests of arterial compliance, carotid atherosclerosis, left ventricular mass, systolic and diastolic function, heart rate variability, a maximal bicycle test with VO_2_ determinations) were recoded. An OGTT with insulin determinations has been made. Whole-body MRI with determinations of visceral/subcutaneous adipose tissue and liver and pancreatic fat has been performed. Fat mass has been recorded by DXA. Also genomic, proteomic, and metabolomics measurements have been analyzed.

**Figure 2.**
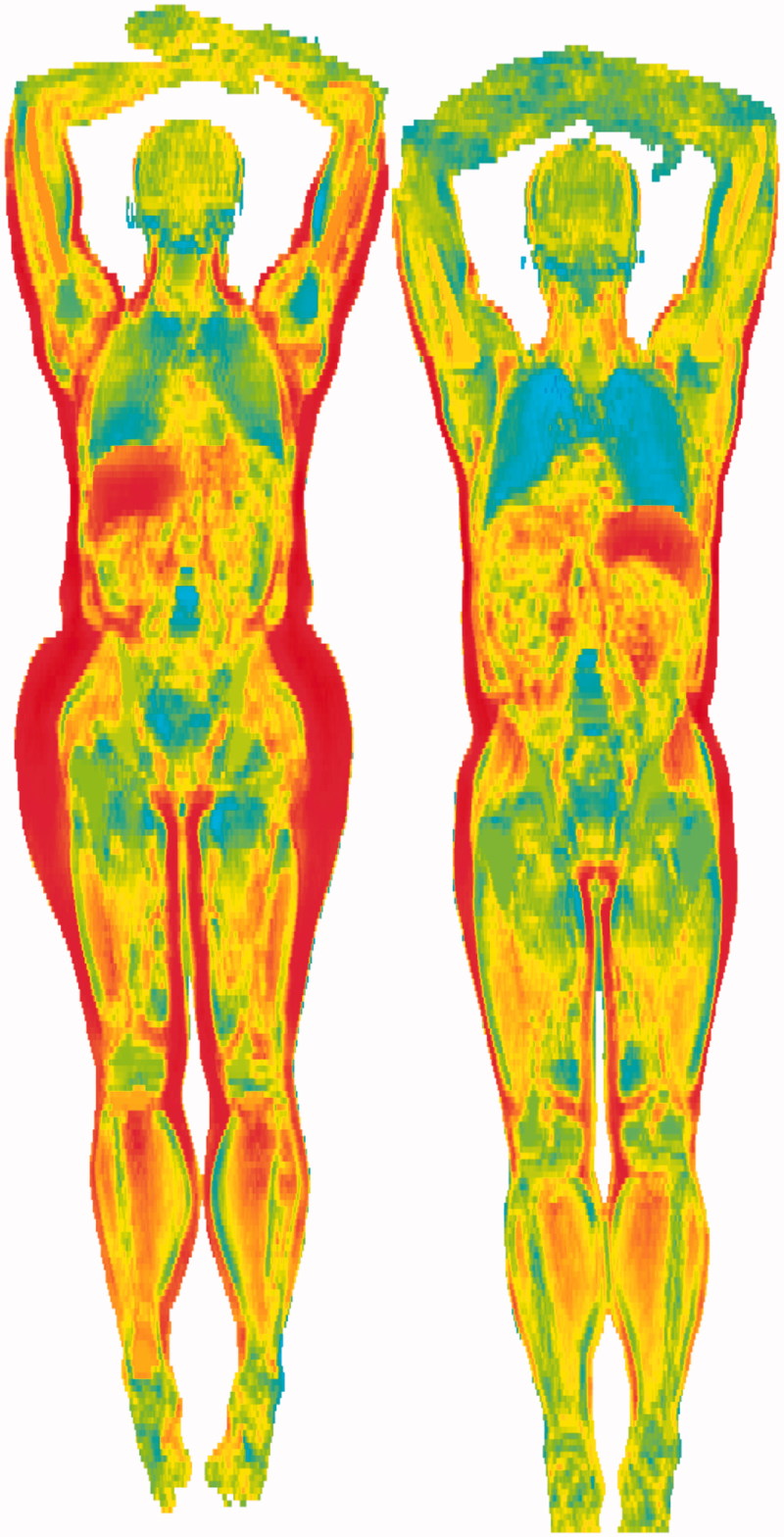
‘Imiomics analysis’ of fat mass measured by bioimpedance in females (left) and males (right). A correlation analysis was performed between fat mass and the relative volume of each image element in the 3D magnetic resonance image. Each image element is then color-coded according to the correlation coefficient, where dark red represents a high positive correlation coefficient and blue a high negative correlation coefficient. Unpublished data from Robin Strand, Joel Kullberg, and Håkan Ahlström at the Department of Radiology, Uppsala University.

Based on data from MRI in POEM, a novel concept to analyze relationships between a phenotype and whole-body images has been developed at the Department of Radiology, called ‘imiomics’ (29). By this approach, each of the >2 million image elements in the 3 D magnetic resonance image is quantified in terms of volume and lipid content, and each image element could be related to any phenotype, and later correlation maps of the body could be constructed in 3 D. Figure 2 shows such an analysis when fat mass measured by bioimpedance is related to the relative volume of each image element.

The major strength of the POEM study is the extremely detailed characterization of metabolism and the cardiovascular system.

## The EpiHealth study

From April 2011, men and women in the age range 45–75 years in two Swedish towns, Uppsala and Malmö, have been invited in a random fashion to an on-going health screening survey, EpiHealth (Epidemiology for Health) (www.epihealth.se) (30). The study was started as a part of the government-supported Strategic Research Areas, and the major aim of the study is to investigate interactions between genes and lifestyle factors regarding common diseases in the elderly.

By 31 December 2017, data on approximately 24,000 individuals have been collected (13,000 individuals in Uppsala). Traditional CV risk factors and fat mass (bioimpedance) have been recorded. Genomic, metabolomic, and proteomic data have been collected in a subsample (*n* = 2500). In spring 2017, incident CV diseases were updated by Swedish in-hospital care and mortality registers.

In a substudy, atherosclerosis has been measured, and a whole-body PET/MRI scan with FDG-tracer has been performed with special reference to inflammation of atherosclerosis in the aorta and carotid arteries (*n* = 100).

The major strength of EpiHealth is the rather large size of the cohort, which will enable a fairly rapid collection of incident CVD endpoint, and provide a good power for ‘omics’ studies.

## The SCAPIS study

As a collaboration project between six Swedish universities, 30,000 subjects aged 50–65 years will be investigated in the Swedish CArdioPulmonary Imaging Study (SCAPIS) with a planned ‘last subject in’ in Q4 2018 (5,000 subjects in Uppsala) (www.scapis.se) (31). The major aims are to discover novel mechanisms and biomarkers for CVD and pulmonary diseases to improve risk stratification and discover new drug targets and preventive strategies.

In addition to the traditional CV risk factors, an extensive imaging program is performed, including CT coronary angiography, CT scanning of lungs and abdomen for visceral/subcutaneous adipose tissue and liver fat (including ‘inflamed adipose tissue’ determinations), as well as ultrasound for carotid artery atherosclerosis (and MRI if carotid plaques are found). A detailed pulmonary function testing battery has been performed together with measurements of the ankle–brachial index to assess leg atherosclerosis. Genomic, proteomic, and metabolomics measurements are planned in a subsample of 5,000 during 2017 and 2018.

The SCAPIS study is the largest academic collaborative project in Sweden in medicine with a budget of >40 million €for collection of data.

**Figure 3.**
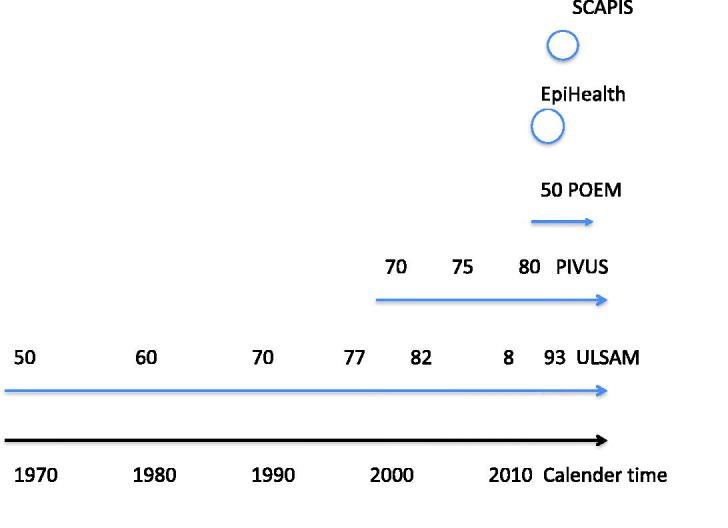
This calendar shows when the different studies and their examination cycles were performed.

Table I summarizes the major phenotypes collected in the CVD studies in Uppsala, and Figure 3 shows the when the studies were performed.

**Table 1.** Table showing which kinds of investigations were performed in the different studies.

	ULSAM	PIVUS	POEM	EpiHealth	SCAPIS
*n* at baseline	2322	1016	503	13000	5000
Traditional risk factors	✓	✓	✓	✓	✓
Fatty acid composition	✓	✓	✓		
DXA	✓	✓	✓		
Dietary data	✓	✓	✓	✓	✓
Echocardiography	✓	✓	✓		
Carotid atherosclerosis	✓	✓	✓		✓
Coronary atherosclerosis					✓
Endothelial function/arterial compliance		✓	✓		
OGTT	✓		✓		
Hyperinsulinemic clamp	✓				
24-h blood pressure	✓		✓		✓
Abdominal MRI/CT		✓	✓		✓
Myocardial MRI		✓			

CT: computer tomography; DXA: dual X-ray absorptiometry; MRI: magnetic resonance imaging; OGTT: oral glucose tolerance test.

## Discussion

Two extremes of epidemiological studies exist. First, studies with a limited number of subjects (some hundreds to a couple of thousands) that have been extensively phenotyped; ULSAM, PIVUS, and POEM are examples of such studies. Second, large high-throughput studies with less deep phenotyping; EpiHealth is one such example. The development of ‘omics’ technologies, especially GWAS, demands large-scale studies to deal with the multiple testing issue. On the other hand, to evaluate mechanisms involved in the development of CVD, the small-scale studies are needed with deep phenotyping of markers of subclinical CVD, such as endothelial function, atherosclerosis, and myocardial function. Thus, both large- and small-scale epidemiological studies are needed in order to move the knowledge of CVD forward. (Table I)

**Figure 4.**
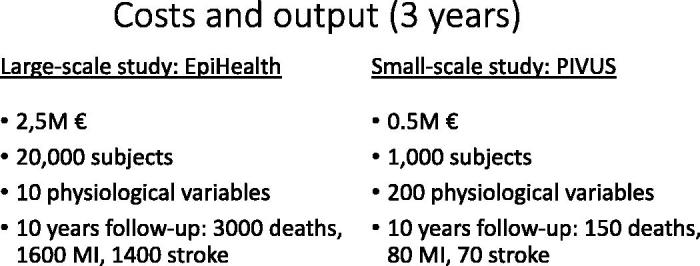
Costs and outcome during three years of data collection in two extremes of epidemiological studies.

Figure 4 illustrates the costs and output in terms of measured phenotypes and outcomes in two examples of large-scale and small-scale Uppsala epidemiological studies.

In the SCAPIS study, the features of both the large- and small-scale studies are merged into a large study with deep phenotyping. However, to accomplish that goal in a reasonable time-frame, this study has to be performed as a multi-center study and at a high cost.

In conclusion, several CVD cohort studies with different characteristics exist in Uppsala which makes it possible to explore hypotheses demanding both a large number of participants, as well as a deep phenotyping of markers of subclinical CVD and long follow-up periods. In addition, having access to several cohorts makes it possible to replicate findings in one sample in another study, an important task to ascertain the validity of novel research findings.

## Disclosure statement

No potential conflict of interest was reported by the authors.
